# Accuracies of genomic prediction for twenty economically important traits in Chinese Simmental beef cattle

**DOI:** 10.1111/age.12853

**Published:** 2019-09-09

**Authors:** B. Zhu, P. Guo, Z. Wang, W. Zhang, Y. Chen, L. Zhang, H. Gao, Z. Wang, X. Gao, L. Xu, J. Li

**Affiliations:** ^1^ Laboratory of Molecular Biology and Bovine Breeding Institute of Animal Sciences Chinese Academy of Agricultural Sciences Beijing 100193 China; ^2^ National Centre of Beef Cattle Genetic Evaluation Beijing 100193 China; ^3^ College of Computer and Information Engineering Tianjin Agricultural University Tianjin 300384 China; ^4^ Department of Agricultural, Food and Nutritional Science University of Alberta Edmonton, AB T6G 2R3 Canada

**Keywords:** accuracy, Bayesian methods, cross‐validation, economic traits, prediction

## Abstract

Genomic prediction has been widely utilized to estimate genomic breeding values (GEBVs) in farm animals. In this study, we conducted genomic prediction for 20 economically important traits including growth, carcass and meat quality traits in Chinese Simmental beef cattle. Five approaches (GBLUP, BayesA, BayesB, BayesCπ and BayesR) were used to estimate the genomic breeding values. The predictive accuracies ranged from 0.159 (lean meat percentage estimated by BayesCπ) to 0.518 (striploin weight estimated by BayesR). Moreover, we found that the average predictive accuracies across 20 traits were 0.361, 0.361, 0.367, 0.367 and 0.378, and the averaged regression coefficients were 0.89, 0.86, 0.89, 0.94 and 0.95 for GBLUP, BayesA, BayesB, BayesCπ and BayesR respectively. The genomic prediction accuracies were mostly moderate and high for growth and carcass traits, whereas meat quality traits showed relatively low accuracies. We concluded that Bayesian regression approaches, especially for BayesR and BayesCπ, were slightly superior to GBLUP for most traits. Increasing with the sizes of reference population, these two approaches are feasible for future application of genomic selection in Chinese beef cattle.

## Introduction

Genomic prediction has been widely utilized to estimate genomic breeding values (GEBVs) for quantitative traits in breeding program of farm animals (Hayes *et al*. 2009; Goddard *et al*. 2010). The application of genomic selection is considered an important revolution for the theory of animal breeding over the past two decades (Hayes *et al*. 2009; Heffner *et al*. 2009; de Los Campos *et al*. 2013; Spelman *et al*. 2013). With the advances of genomic selection technologies, this strategy has led to dramatic increases in genetic progress in farm animals (Goddard *et al*. 2016; Kumar & Hedges 2016). For instance, rates of genetic gain per year for US Holstein increased from about 50% to 100% for yield traits and from threefold to fourfold for lowly heritable traits (Garcia‐Ruiz *et al*. 2016).

In beef cattle, genomic prediction offers great promise to predict genetic merits of selection candidates, especially for traits that are difficult or expensive to measure, such as carcass merit traits. The success of genomic selection depends on the accuracies of GEBVs, which are largely affected by the predictive approaches, the size of reference population, trait heritability and the extent of the linkage disequilibrium between SNPs and QTL (Hayes *et al*. 2009; VanRaden *et al*. 2009). Many studies have assessed the predictive accuracies of GEBVs for economically important traits in different beef cattle populations using the BovineSNP50 BeadChip (Saatchi *et al*. 2011, 2012; Todd *et al*. 2014; Chen *et al*. 2015), and their results show varying degrees of accuracy for GEBVs. For instance, genomic prediction for growth, meat quality and reproduction traits in US Limousin and Simmental beef cattle revealed accuracies of GEBVs ranging from 0.39 to 0.76 and 0.29 to 0.65 respectively(Saatchi *et al*. 2012). Accuracies of GEBVs for US Angus ranged from 0.22 to 0.69 (Saatchi *et al*. 2011). Using genomic best linear unbiased prediction (GBLUP) and BayesB methods, the accuracies of genomic prediction for carcass traits in Canadian Angus and Charolais cattle varied from 0.16 to 0.6 (Chen *et al*. 2015). Using simulation studies of carcass traits in UK Limousin, terminal index accuracy of GEBVs varied from 0.18 to 0.73 (Todd *et al*. 2014). Only a few studies have evaluated the predictive accuracies of multiple methods using the Illumina BovineHD chip. For example, Neves *et al*. (2014) and Fernandes Jr. *et al*. (2016) evaluated genomic prediction for growth and carcass traits of Nellore cattle using GBLUP, BayesC and Bayesian Lasso methods. Their findings showed that the predictive accuracies varied among traits using different approaches.

Chinese Simmental is one of the predominant beef cattle in China (representing approximately 70% of beef market), which is particularly renowned for rapid growth rate and palatable meat quality (Niu *et al*. 2016). Previous studies have comprehensively investigated the molecular mechanisms underlying important traits, such as foreshank weight, triglyceride levels and shear force, using genome‐wide association studies (Wu *et al*. 2014; Xia *et al*. 2016; Zhang *et al*. 2016). Based on this population, genomic prediction using Bayesian regression methods with variable degrees of freedom and scale parameters have been evaluated for live weight and tenderloin weight (Zhu *et al*. 2016). However, until now, no studies have evaluated the accuracies of genomic prediction for economically important traits such as growth, carcass (especially for retail beef cuts) and meat quality traits using multiple methods in Chinese Simmental cattle. The objective of this study was to estimate and compare the predictive accuracies and abilities of GEBVs for 20 traits, including growth, carcass and meat quality, using five methods (GBLUP, BayesA, BayesB, BayesCπ and BayesR) in Chinese Simmental beef cattle.

## Materials and methods

### Ethics statement

All animals were treated following the guidelines for the experimental animals established by the Council of China. Protocols of the experiments were approved by the Science Research Department of the Institute of Animal Science, Chinese Academy of Agricultural Sciences (CAAS) (Beijing, China).

### Animals and phenotypes

Animals originated from five farms in Ulgai Grassland, Xilingole League, Inner Mongolia of China. All animals were born between 2008 and 2013 and were weaned at approximately six months of age. After weaning, animals were moved to Beijing Jinweifuren farm for fattening and raised under the same feeding conditions. Animal were measured for growth traits every six or 12 months until slaughter. Live weight was measured after 24 h of fasting. Slaughter age ranged from 18 to 24 months. After slaughter, carcass traits and meat quality traits were measured according to the Institutional Meat Purchase Specification for fresh beef guidelines and GB/T 27643‐2011. In this study, a total of 20 traits were measured and analyzed (Table 1): (i) growth traits: average daily gain (ADG; kg) was calculated by subtracting the entering farm weight from the live weight and dividing by the number of days spent in the farm, and live weight (LW; kg) was measured before slaughter with fasting 24 h; (ii) carcass traits: hot carcass weight (CW; kg), dressing percentage (DP; %), lean meat percentage (LMP; %), back fat thickness (BFT; mm) and retail beef cuts including striploin (ST; kg), spencer roll (SR; kg), chuck roll (CR; kg), tenderloin (TD; kg), fore shank (FS; kg), conical muscle (CM; kg), outside (OU; kg), Silverside (SI; kg), knuckle (KN; kg), inside cap off (ICO; kg), hind shank (HS; kg) and retail meat weight (RMW; kg); and (iii) meat quality traits: potential of hydrogen (pH) and shear force (SF, kg).

**Table 1:** Summary statistics of 20 traits including abbreviation, number of animals, mean, standard deviation (SD), minimum, maximum and definition of 20 traits

Trait	Number	Mean	SD	Maximum	Minimum	Trait definition
ADG	1294	0.97	0.22	2.41	0.38	Average daily gain weight, kg
LW	1302	505.26	70.73	776.00	318.00	Live weight, kg
CW	1302	271.35	45.63	486.00	162.60	Carcass weight, kg
DP	1301	53.56	2.91	68.98	41.03	Dressing percentage, %
LMP	1301	45.47	3.08	61.56	32.51	Lean meat percentage, %
ST	1298	8.68	1.98	15.90	3.21	Striploin, kg
SR	1298	10.70	2.22	18.32	5.03	Spencer roll, kg
CR	1298	11.65	3.25	28.68	4.50	Chuck roll, kg
TD	1299	3.98	0.71	7.84	2.20	Tenderloin, kg
FS	1298	5.02	0.92	10.90	2.94	Fore shank, kg
CM	1158	1.07	0.19	2.20	0.60	Conical muscle, kg
OU	1299	15.06	2.33	23.60	7.88	Outside, kg
SI	1299	13.18	2.38	23.72	7.70	Silverside, kg
KN	1299	9.57	1.48	14.40	6.18	Knuckle, kg
ICO	1299	11.89	2.08	20.98	7.12	Inside cap off, kg
HS	1300	8.03	1.19	12.12	4.84	Hind shank, kg
RMW	1299	169.94	29.8	280.87	84.00	Retail meat weight, kg
BFT	654	2.70	2.01	13.40	0.05	Back fat thickness, mm
pH	1255	5.64	0.38	7.16	4.00	Potential of hydrogen
SF	1272	5.53	1.94	13.14	1.33	Shear force, kg

### Genotype and quality control

A total of 1302 Simmental beef cattle were genotyped with the Illumina BovineHD SNP array. Missing SNPs were imputed using beagle v3.3.1 software (Browning & Browning 2007). Prior to statistical analysis, genotypes were edited using plink v1.07 software (Purcell *et al*. 2007) for the following: (i) minor allele frequency (>0.05), (ii) proportion of missing genotypes (<0.05) and (iii) Hardy‐Weinberg equilibrium (*P* > 10^−6^). SNPs satisfying these criteria were used to interrogate the linkage disequilibrium with syntenic SNPs located with a window of 100 neighboring markers, which resulted in only one SNP form each pair of highly correlated SNPs (*r* ^2^ > 0.995) remaining in the SNP dataset. After these quality controls, the final data consisted of 1217 individuals and 459 268 filtered SNPs in the autosomes. Genotype data are available from the Dryad Digital Repository (https://doi.org/10.5061/dryad.4qc06).

### Statistical model and genetic analyses

Heritabilities were calculated using a restricted maximum likelihood method with an animal model in asreml v3.0 software (Gilmour *et al*. 2009). Relationships between animals were estimated using a G matrix, where G was the genomic relationship matrix and inferred from SNP markers, as suggested by VanRaden (2008). The animal model included random additive polygenic effects, fixed effects and residual for all traits. The additive polygenic effects were treated as random and assumed to be mutually independent. Fixed effects in the model included gender, farm and year of measurement. In addition, animals' age at slaughter were considered covariates in the model except for average daily gain traits.

To estimate GEBVs, we used the following five statistical methods: (i) (GBLUP) (VanRaden 2008) and (ii) Bayesian regression using mixture models, including BayesA and BayesB (Meuwissen *et al*. 2001), BayesCπ (Habier *et al*. 2011) and a variable selection method BayesR (Erbe *et al*. 2012). These methods were compilied using C language (Zhu *et al*. 2016).

In all cases, the statistical model used was the following:

(1) y=Xb+Zg+e

### GBLUP

For GBLUP, **y** is an *N* × 1 (*N* = number of observations) vector of phenotype in Equation (1), **X** is an incidence matrix of the fixed effects, **Z** is the incidence matrix allocating records to GEBVs, **g** is the vector of GEBVs and **e** is a vector of residuals. It is assumed that **g** follows a normal distribution $N(0,G\sigma_{g}^{2})$. Given **b** and **g**,** y** is conditionally independent and distributed as: $\left(y|b,g,\sigma_{e}^{2}\right)∼N\left(\mathrm{Xb}+\mathrm{Zg},I\sigma_{e}^{2}\right)$

### BayesA, BayesB and BayesCπ

The Bayesian model used the same equation as in (1), where **y**,** X**,** b** and **e** were defined as before, but **g** represented an *M* × 1 vector of SNP marker effects; **Z** is an *N* × *M* matrix of SNPs, coded with values 0, 1 or 2 for genotypes 11, 12 and 22 respectively; and *Z*_*ij*_ denotes marker *j* of individuals *i*.

The prior for ***g***_*j*_ depends on the variance $\sigma_{j}^{2}$ and the prior probability *π*. In BayesA, all SNPs have effects, i.e. $\sigma_{j}^{2}∼x^{-2}\left(v,vS_{a}^{2}\right)$, and *π* is equal to 0. $\sigma_{j}^{2}$ denotes that SNP *j* has its own variance, with the parameters of ***v*** and $S_{a}^{2}$. In BayesB, the two‐component mixture, with one component being $t(0,v,S_{a}^{2})$ and the other component being a spike at 0, are provided as:

$$\alpha_{j}|\sigma_{j}^{2}∼\left(\text{idd}\right)\left\{\begin{array}{cc} 0 & \text{with probability}\;\pi \\ N(0,\sigma_{j}^{2}) & \text{with probability}\;(1-\pi ) \end{array}\right.$$

where *j* = 1, …… , *P*

Here, *π* represents the proportion of SNPs with no genetic effects on the trait of interest. $S_{a}^{2}$ is derived using following equation, $S_{a}^{2}=\frac{\left(v-2\right)*E\left(\sigma_{j}^{2}\right)}{v}$, where *v* is 4.2, as reported by Meuwissen *et al*. (2001). In BayesCπ, the SNP effects have a common variance, $\sigma_{j}^{2}=\sigma_{g}^{2}\left(j=1,\ldots ,P\right)$, and $\sigma_{g}^{2}$ follows a scaled inverse *χ* ^2^ prior with parameters *v* and $S_{a}^{2}$. As a result, the SNP effects with probability (1 − *π*) follows a mixture of multivariate Student's *t*‐distributions $t(0,v,{\text{IS}}_{a}^{2})$. The *π* parameter is treated as unknown with a uniform (0,1) prior distribution.

### BayesR

BayesR is an extension of BayesCπ, where SNP effects are assumed to be sampled from a mixture of normal distributions (Erbe *et al*. 2012; Bolormaa *et al*. 2013). The variance of each component of the mixture is fixed (0, 0.01%, 0.1% or 1% of the genetic variance). The number of SNPs belonging to each component of the mixture is assumed to come from a multinomial distribution with proportions *p*_*i*_ (*i *=* *1, 2, 3 or 4) in which the *p*_*i*_ is drawn from a Dirichlet distribution (a multivariate generalization of a beta distribution) with pseudo‐counts of 1 for each component of the mixture. Thus, the prior assumes that the four components of the mixture are equally probable but with minimal prior knowledge of these probabilities.

### SNP effects estimation

SNP effects were estimated using the Markov chain Monte Carlo sampling algorithm in BayesA, BayesB, BayesCπ and BayesR. Markov chains were run for 50 000 cycles of Gibbs sampling. The first 10 000 were discarded as burn‐in. Then GEBVs were calculated as GEBV_*i*_ = ∑*Z*_*ij*_ *α*_*j*_.

### Validation of the models

To assess the predictive accuracies for 20 economically important traits, we used a five‐fold cross‐validation method (Luan *et al*. 2009). Overall, 1217 individuals were divided into reference and validation populations. The genotyped individuals were randomly divided into five groups, whereas phenotypes of animals in validation set were masked to be unknown. Thus, 974 individuals were randomly sampled as the reference set, and the remaining 243 individuals as the validation set. The whole procedure was repeated 10 times.

### Comparison criteria

Three methods were utilized to evaluate the predictive ability based on comparison of GEBVs with corrected phenotypes of animals in the validation population:

The correlations between GEBV and corrected phenotype were calculated to evaluate the predictive ability ($r_{\mathrm{GEBV},\hat{y}}$). To remove the influence of heritability on the predictive ability, Pearson's correlation between GEBV and corrected phenotype was divided by the square root of heritability ($r_{\mathrm{GEBV},\hat{y}}/\sqrt{h^{2}}$), where $\hat{y}$ was the corrected phenotype. This value is approximate to the correlation between the true breeding value and GEBVs (Pryce *et al*. 2012).
The slope of the regression of $\hat{y}$ on GEBV for animals in the validation population ($b_{\hat{y},\mathrm{GEBV}}$) was calculate to measure the degree of inflation or deflation of genomic prediction. Estimates of $b_{\hat{y},\mathrm{GEBV}}$ close to 1 are indicative of predictions that are similar to that of corrected phenotype on scale.
The mean squared error of prediction (MSE) between $\hat{y}$ and GEBV in the validation population was used to measure the overall fit of model, and the computation equation was $\text{MSE}=\frac{1}{N}\sum \left(\text{GEBV}-\hat{y}\right)$, where *N* is the number of individuals. A large estimated value of predictive accuracy is indicative of reliable prediction, and a low MSE value means a better overall fit.

## Results

### Heritability estimates

Heritability estimates for 20 traits ranged from 0.04 to 0.62. We found that 10 traits showed relatively high heritabilities: FS (0.4), LW (0.43), RMW (0.43), ADG (0.47), BI (0.47), ICO (0.51), ER (0.52), OU (0.6), HS (0.61) and KN (0.62). Eight traits showed moderate heritabilities— BFI (0.21), ST (0.24), SR (0.26), CR (0.27), DP (0.31), CW (0.38), LMP (0.39) and TD (0.39)—whereas three traits had low heritabilities—pH (0.04), BFT (0.1) and SF (0.15). The standard errors for all heritability estimates were less than 0.04 (Table 2).

**Table 2:** Estimates of variance components and heritability with standard errors for twenty traits

Trait	$\sigma_{a}^{2}$	$\sigma_{e}^{2}$	*h* ^2^
ADG	0.012	0.013	0.47 ± 0.03
LW	663.380	1139.880	0.37 ± 0.02
CW	299.650	361.820	0.45 ± 0.02
DP	0.950	4.960	0.16 ± 0.02
LMP	0.850	5.110	0.14 ± 0.01
ST	0.380	1.160	0.24 ± 0.02
SR	0.490	1.390	0.26 ± 0.01
CR	1.080	2.880	0.27 ± 0.02
TD	0.078	0.120	0.39 ± 0.03
FS	0.150	0.220	0.40 ± 0.02
CM	0.008	0.009	0.47 ± 0.02
OU	1.320	0.880	0.60 ± 0.04
ER	1.200	1.130	0.52 ± 0.03
KN	0.590	0.360	0.62 ± 0.04
ICO	0.910	0.880	0.51 ± 0.03
HS	0.370	0.230	0.61 ± 0.02
RMW	126.130	169.570	0.43 ± 0.02
BFT	0.330	2.940	0.10 ± 0.01
pH	0.005	0.112	0.04 ± 0.01
SF	0.340	2.000	0.15 ± 0.01

$\sigma_{a}^{2}$, additive genetic variance; $\sigma_{e}^{2}$, environmental variance; *h* ^2^, heritability ± standard error; ADG, BFT, back fat thickness; CM, conical muscle; CR, chuck roll; CW, carcass weight; DP, dressing percentage; FS, fore shank; ICO, inside cap off; HS, hind shank; KN, knuckle; LMP, lean meat percentage; LW, live weight; OU, outside; pH, potential of hydrogen; RMW, retail meat weight; SF, shear force; SI, silverside; SR, spencer roll; ST, striploin; TD, tenderloin.

### Predictive ability of five methods

We evaluated the predictive abilities for these 20 traits using different methods based on 5‐fold cross‐validation. The summary of predictive results is presented in Table 3. Overall, we observed that the predictive abilities ($r_{\mathrm{GEBV},\hat{y}}$) ranged from 0.059 (for LMP estimated by BayesCπ) to 0.376 (for HS estimated by BayesB; Table 3). The average predictive abilities across 20 traits were 0.216, 0.216, 0.221, 0.220 and 0.225 for GBLUP, BayesA, BayesB, BayesCπ and BayesR respectively.

**Table 3:** Predictive abilities and accuracies for 20 traits in Chinese Simmental beef cattle based on five approaches using five‐fold cross‐validation

Trait	$r_{\mathrm{GEBV},\hat{y}}$					$r_{\mathrm{GEBV},\hat{y}}/\sqrt{h^{2}}$				
	GBLUP	BayesA	BayesB	BayesCπ	BayesR	GBLUP	BayesA	BayesB	BayesCπ	BayesR
ADG	0.194	0.197	0.197	0.204	0.214	0.283	0.287	0.288	0.298	0.312
LW	0.231	0.232	0.230	0.239	0.242	0.379	0.381	0.378	0.393	0.398
CW	0.251	0.253	0.252	0.261	0.268	0.374	0.377	0.376	0.389	0.400
DP	0.111	0.111	0.112	0.109	0.119	0.277	0.276	0.279	0.273	0.298
LMP	0.061	0.061	0.061	0.059	0.069	0.162	0.164	0.162	0.159	0.184
ST	0.239	0.238	0.241	0.239	0.254	0.487	0.486	0.492	0.487	0.518
SR	0.178	0.179	0.176	0.177	0.184	0.349	0.352	0.345	0.348	0.361
CR	0.168	0.169	0.169	0.169	0.176	0.322	0.326	0.324	0.325	0.339
TD	0.277	0.278	0.278	0.283	0.291	0.444	0.446	0.446	0.453	0.466
FS	0.249	0.250	0.254	0.250	0.252	0.394	0.395	0.402	0.395	0.398
CM	0.240	0.240	0.250	0.247	0.251	0.35	0.351	0.365	0.360	0.366
OU	0.346	0.347	0.347	0.352	0.358	0.447	0.448	0.447	0.455	0.462
ER	0.354	0.353	0.355	0.358	0.361	0.491	0.49	0.492	0.497	0.501
KN	0.315	0.304	0.339	0.312	0.311	0.400	0.385	0.431	0.396	0.395
ICO	0.258	0.259	0.259	0.269	0.268	0.362	0.363	0.363	0.377	0.375
HS	0.325	0.326	0.376	0.332	0.331	0.416	0.417	0.481	0.425	0.424
RMW	0.267	0.268	0.262	0.271	0.278	0.408	0.409	0.399	0.414	0.424
BFT	0.074	0.072	0.074	0.076	0.077	0.235	0.227	0.233	0.241	0.243
pH	0.073	0.073	0.073	0.074	0.078	0.366	0.365	0.365	0.370	0.390
SF	0.109	0.107	0.107	0.114	0.119	0.280	0.277	0.277	0.294	0.307

Note: Predictive ability ($r_{\mathrm{GEBV},\hat{y}}$) was calculated by the correlation between GEBV and corrected phenotype. Predictive accuracy ($r_{\mathrm{GEBV},\hat{y}}/\sqrt{h^{2}}$) was computed as Pearson's correlation between GEBV and corrected phenotype divided by square root of heritability.

ADG, average daily gain; BFT, back fat thickness; CM, conical muscle; CR, chuck roll; CW, carcass weight; DP, dressing percentage; FS, fore shank; HS, hind shank; ICO, inside cap off; KN, knuckle; LMP, lean meat percentage; LW, live weight; OU, outside; pH, potential of hydrogen; RMW, retail meat weight; SF, shear force; SI, silverside; SR, Spencer roll; ST, striploin; TD, tenderloin.

**Figure 1:**
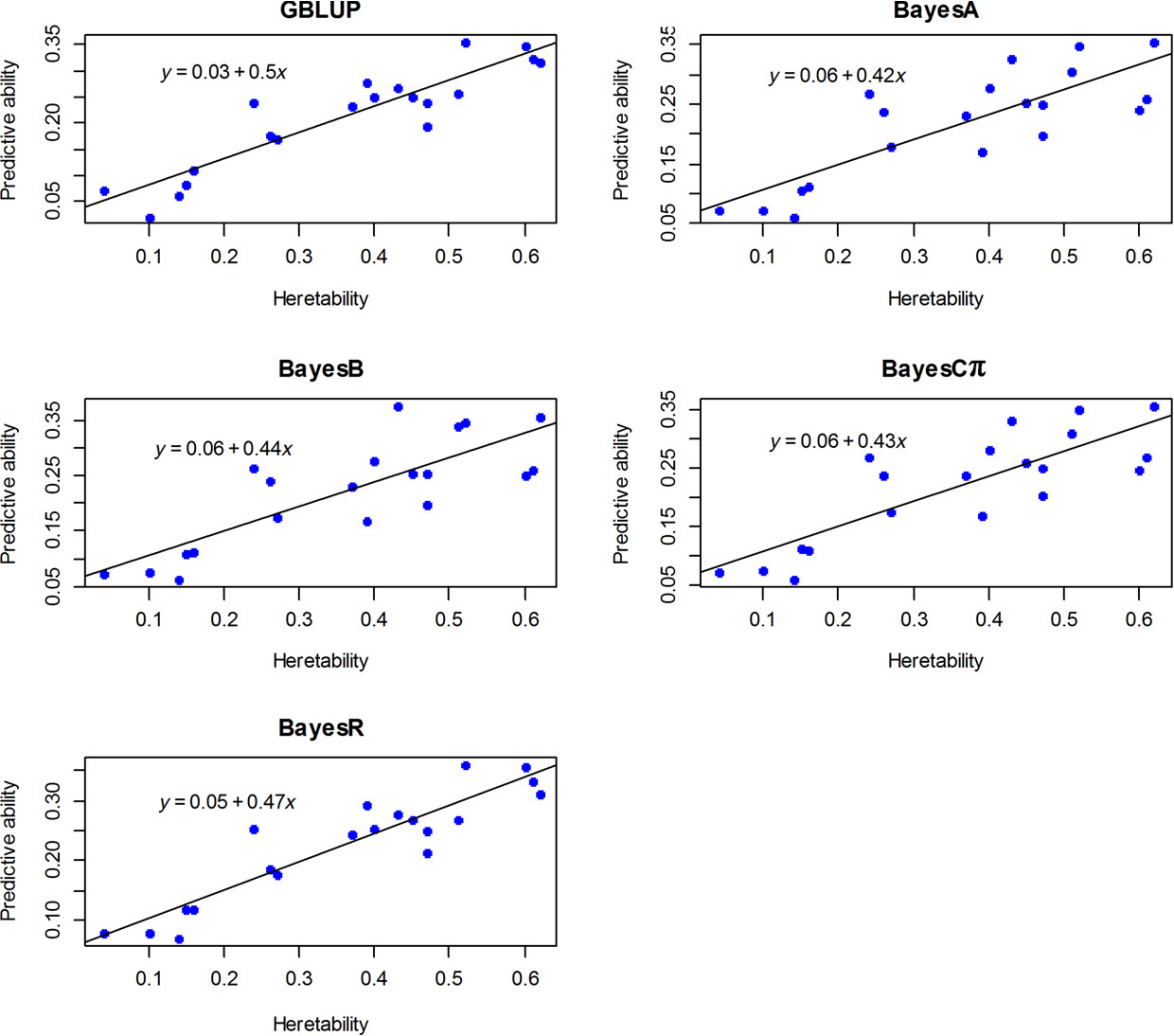
Regression of predictive ability on heritability for 20 traits using five statistics methods.

To investigate the relationship between predictive ability and trait heritability, we next estimated the regression coefficient of predictive ability on heritability for these methods. Our results showed that the predictive abilities were linearly correlated with trait heritability. The strongest correlation (0.5) was observed across 20 traits using GBLUP compared with Bayesian methods (Fig. 1). Among Bayesian methods, we found the highest regression coefficient of predictive ability on heritability was 0.47 in BayesR.

### Predictive accuracy of five methods

After removing the influence of heritability from predictive ability, we found obvious difference in predictive accuracies ranging from 0.159 (LMP) to 0.518 (ST). The average predictive accuracies across traits were 0.361 for GBLUP, 0.361 for BayesA, 0.367 for BayesB, 0.367 for BayesCπ and 0.378 for BayesR respectively (Table 3). In general, we observed the predictive accuracies of BayesA were consistent with those of GBLUP for most traits, whereas other Bayesian regression methods (BayesB, BayesCπ and BayesR) showed slightly higher accuracies than did GBLUP. The advantage of Bayesian regression methods over GBLUP was obtained for HS (6.5%), CW (1.5%) and ST (3.1%) using BayesB, BayesCπ and BayesR. Conversely, GBLUP was slightly superior to BayesA, BayesB, BayesCπ and BayesR for KN (1.5%), RMW (0.9%), KN (0.4%) and KN (0.5%) in the present study (Table 3).

### Scale of genomic predictions and mean squared prediction error

The regression coefficient of corrected phenotype on GEBV was calculated as a measurement of the bias for the prediction. In this study, we observed that predictions of GEBV using both GBLUP and Bayesian regression methods were inflated for most traits, whereas for traits ST, TD and  pH, the predictions from GBLUP and Bayesian regression methods tended to be slightly deflated (Table 4). We found that predictions of GEBV using GBLUP, BayesA and BayesB were inflated for LW and RMW, whereas those from BayesCπ and BayesR were slightly deflated. The average regression coefficients across traits were 0.89, 0.86, 0.89, 0.94 and 0.95 for GBLUP, BayesA, BayesB, BayesCπ and BayesR respectively.

**Table 4:** Inflation and mean squared error (MSE) of genomic prediction for 20 traits in Chinese Simmental beef cattle based on five approaches using five‐fold cross‐validation

Trait	*b*($\hat{y}$,GEBV)					MSE				
	GBLUP	BayesA	BayesB	BayesCπ	BayesR	GBLUP	BayesA	BayesB	BayesCπ	BayesR
ADG	0.63	0.67	0.70	0.93	0.96	0.16	0.17	0.17	0.16	0.15
LW	0.84	0.85	0.83	1.08	1.04	45.48	47.16	46.21	46.51	46.54
CW	0.72	0.74	0.76	0.94	0.95	27.26	29.99	29.79	28.73	28.94
DP	0.87	0.88	0.91	0.65	0.88	2.34	2.40	2.41	2.54	2.48
LMP	0.28	0.31	0.27	0.47	0.43	2.44	2.49	2.47	2.42	2.46
ST	1.56	1.58	1.59	1.76	1.79	19.00	19.41	19.44	22.08	20.12
SR	0.82	0.82	0.86	0.85	0.88	1.42	1.45	1.45	1.45	1.45
CR	0.59	0.61	0.62	0.70	0.76	1.55	1.55	1.55	1.55	1.56
TD	1.01	1.04	1.09	1.19	1.21	2.36	2.42	2.37	2.36	2.35
FS	0.94	0.95	0.97	0.98	0.99	0.49	0.49	0.53	0.49	0.48
BI	0.96	1.00	1.07	1.27	1.25	0.88	0.88	0.89	0.88	0.87
OU	0.83	0.84	0.87	0.92	0.94	0.18	0.20	0.19	0.19	0.18
ER	0.78	0.77	0.78	0.81	0.87	1.65	1.84	1.92	1.91	1.89
KN	0.67	0.67	0.77	0.78	0.79	1.65	1.64	2.12	1.75	1.96
ICO	0.70	0.72	0.74	0.90	0.85	1.10	1.11	1.45	1.12	1.35
HS	0.75	0.76	0.86	0.87	0.89	1.41	1.41	1.55	1.40	1.48
RMW	0.94	0.95	0.97	1.15	1.02	0.86	0.87	0.84	0.92	0.89
BFT	0.45	0.37	0.45	0.50	0.48	0.96	0.96	0.96	0.96	0.97
pH	1.98	1.95	2.08	1.33	1.32	1.71	1.71	1.75	1.72	1.74
SF	1.39	0.64	0.63	0.79	0.76	1.30	1.30	1.31	1.31	1.31

ADG, average daily gain; BFT, back fat thickness; CM, conical muscle; CR, chuck roll; CW, carcass weight; DP, dressing percentage; FS, fore shank; HS, hind shank; ICO, inside cap off; KN, knuckle; LMP, lean meat percentage; LW, live weight; OU, outside; pH, potential of hydrogen; RMW, retail meat weight; SF, shear force; SI, silverside; SR, Spencer roll; ST, striploin; TD, tenderloin.

For most traits, we found GBLUP generally outperformed Bayesian regression methods based on the MSE (Table 4). However, for ADG, LMP, TD, FS, BI, KN, HS and RMW, lower estimates of MSE were obtained for Bayesian methods.

## Discussion

Previous studies have been conducted for genomic prediction in multiple breeds including Angus, Limousin, Simmental, Charolais, Hereford, Japanese Black, Nellore and other crossbreds using BovineSNP50 and BovineHD SNP arrays (Saatchi *et al*. 2011, 2012; Bolormaa *et al*. 2013; Akanno *et al*. 2014; Gunia *et al*. 2014; Hulsman Hanna *et al*. 2014; Onogi *et al*. 2014; Todd *et al*. 2014; Rolf *et al*. 2015; Fernandes Jr. *et al*. 2016). Su *et al*. (2012) have investigated the difference of predictive accuracies between the BovineHD array and BovineSNP50 using the GBLUP method, and they found that the reliability of GEBV for protein, fertility and udder health traits using the BovineHD array was higher (0.5–1%) than that of 54K array in Holstein.

We previously investigated the pattern of linkage disequilibrium using the BovineHD SNP array in Chinese Simmental cattle, and our findings suggested that the high density SNP array was sufficient to achieve high accuracy for genomic prediction in Chinese Simmental population (Niu *et al*. 2016). To our knowledge, this study is the first attempt to investigate the performance of genomic prediction for 20 economically important traits using BovineHD SNP arrays in Chinese Simmental beef cattle.

### Comparisons of genomic prediction methods

In the current study, we found that the Bayesian regression approaches performed better than did GBLUP for most traits. Based on the estimations of predictive accuracies and regression coefficients, Bayesian regression methods were superior to GBLUP, whereas GBLUP had smaller MSE compared to Bayesian regression approaches.

Previous studies suggested the superiority of Bayesian regression approaches over GBLUP when the number of SNPs is larger than the genotyped animals, i.e. several simulation studies revealed that Bayesian regression approaches have higher accuracies than does GBLUP (Meuwissen *et al*. 2001; Habier *et al*. 2007; Solberg *et al*. 2008; Clark *et al*. 2011). These findings were also consistent with many previous studies using real data (Erbe *et al*. 2012; Pryce *et al*. 2012; Gunia *et al*. 2014; Neves *et al*. 2014; Fernandes Jr. *et al*. 2016). In this study, we found that genomic predictions using Bayesian regression approaches were superior to that of GBLUP (Table 3). This can be explained by the fact that the assumption of Bayesian approaches is more suitable for fitting the genetic architecture of quantitative trait (Rolf *et al*. 2015). However, most of the Bayesian regression approaches, except for BayesA, showed high prediction accuracies for these 20 traits. A large number of SNPs with small effects in BayesA are likely to cause noise for the estimation of the GEBVs (Habier *et al*. 2011). In contrast, Bayesian approaches like BayesB, BayesCπ and BayesR assume that only a small proportion of markers have effects, which may avoid the potential bias caused by linkage disequilibrium (Erbe *et al*. 2012).

Moreover, we observed higher predictive accuracies using BayesR for most of the traits, which implies the segregation of genes with larger effects for them. For traits with mutations of moderate effect segregating and a high number of significant SNPs, a recent study showed that the accuracy of GEBVs with BayesR was higher than with GBLUP (Bolormaa *et al*. 2013). In our study, the average predictive accuracies of 20 traits for BayesR increased ~1.7% compared to GBLUP. Among 20 traits, the highest increase in accuracy of BayesR over GBLUP was observed for ADG (2.9%) and ST (3.1%). We also identified several SNPs with large effects for ADG using BayesR. These SNPs had been previously identified as significant associated SNPs in the gene *NCAPG* and can explain ~4.01% of the phenotypic variances (Zhang *et al*. 2016).

### Predictive abilities and accuracies

The accuracy of GEBVs can be affected by trait heritabilities, size of training population, and breed and statistical method (Bolormaa *et al*. 2013). For example, traits with high heritability (*h* ^2^) and a large training population (*n* = *T*) give higher accuracies, which can be expected from the theory that *Th* ^2^ is a critical parameter (de Roos *et al*. 2008). In our study, we found that Bayesian regression approaches outperformed GBLUP in predictive accuracies for most traits. Moreover, the accuracy of GEBVs for several important traits varied in other cattle populations. For instance, Neves *et al*. (2014) presented the results of implementation of genomic prediction for weight and carcass traits, gestation length and scrotal circumference traits in 685 Nellore cattle. They found that the average accuracy was 0.39 for GBLUP and 0.44 for both BayesC and Bayesian LASSO methods, which was higher than in our study. Under data‐splitting strategies by birth year, Chen *et al*. (2015) accessed the predictive accuracies for hot carcass weight via PBLUP (0.33 for Angus, 0.42 for Charolais), GBLUP (0.34 for Angus, 0.18 for Charolais) and BayesB (0.34 for Angus, 0.21 for Charolais) using the BovineSNP50 Beadchip. Using five‐fold cross‐validation, we found that the predictive accuracy of carcass weight with Bovine HD SNP array was higher than those in Chen *et al*.'s (2015) study. Also, Bolormaa *et al*. (2013) found that the accuracy of genomic prediction for ADG and CW using the GBLUP approach in multiple beef cattle populations were 0.21 and 0.27. Their results also suggested relatively low accuracy compared to the results in our study (ADG, 0.312 and CW, 0.40). Using *K*‐means clustering validation, the predictive abilities of CW, BFT and SF were 0.59, 0.29 and 0.53 in 2703 registered Simmental beef cattle (Saatchi *et al*. 2012). Their results revealed higher predictive ability for CW, BFT and SF compared with our study, and this finding is likely to be explained by the large population utilized in their study. In addition, other studies have performed genomic prediction for the ADG, CW, SF and BFT traits in diverse populations (Akanno *et al*. 2014; Rolf *et al*. 2015; Fernandes Jr. *et al*. 2016). However, no study has been reported for genomic prediction of DP, LMP and primal cuts in beef cattle.

### Scale of genomic predictions

The scale of predictions is an important factor in determining whether GEBVs can be used for genetic evaluation. For instance, the results of one previous study suggested that overestimation of the genetic merit may cause potential exaggeration of GEBVs compared with traditional EBVs when both progeny‐tested and genomic selection were used for selecting candidates (Vitezica *et al*. 2011). In our study, the average regression coefficients across traits were 0.89, 0.86, 0.89, 0.94 and 0.95 for GBLUP, BayesA, BayesB, BayesCπ and BayesR respectively (Table 4). This finding indicates that the BayesR and BayesCπ approaches generate more reliable predictions for these traits in Chinese Simmental population.

The estimation of scale of genomic predictions may vary with population, genetic inherent of the studied trait and the statistical approaches (Neves *et al*. 2014). Among our studied traits in Chinese Simmental beef cattle, 13 traits generated inflated prediction using GBLUP and Bayesian approaches. Many studies showed a similar inflation trend of genomic prediction in Nellore, Nellore‐Angus crossbred, Holstein and Jersey populations using Bayesian approaches (Duchemin *et al*. 2012; Erbe *et al*. 2012; Hulsman Hanna *et al*. 2014), whereas other studies revealed opposite trends in American Angus using GBLUP and in French Holstein and Montbeliarde populations using Bayesian approaches (Saatchi *et al*. 2011; Colombani *et al*. 2013).

## Conclusions

Using multiple methods (GBLUP, BayesA, BayesB, BayesCπ and BayesR), we conducted genomic prediction for economically important traits including growth, carcass (especially on retail beef cuts) and meat quality traits in Chinese Simmental cattle. Bayesian regression approaches, especially BayesR and BayesCπ, were superior to GBLUP for most traits. Thus, it may be feasible to apply these approaches for genomic prediction of these economically important traits in Chinese Simmental beef cattle. Further improvements are required to enlarge population size and reduce inflation of predictions. In addition, our findings provide valuable insights for further implementation of genomic selection in the commercial beef industry.

## Conflict of interest

The authors have no conflict of interest to declare.

## Data Availability

Datasets are available from the Dryad Digital Repository (https://doi.org/10.5061/dryad.4qc06).
